# Cognitive Status Correlates with CXCL10/IP-10 Levels in Parkinson's Disease

**DOI:** 10.1155/2014/903796

**Published:** 2014-10-15

**Authors:** Natália Pessoa Rocha, Paula Luciana Scalzo, Izabela Guimarães Barbosa, Mariana Soares Souza, Isabela Boechat Morato, Érica Leandro Marciano Vieira, Paulo Pereira Christo, Antônio Lúcio Teixeira, Helton José Reis

**Affiliations:** ^1^Laboratório Interdisciplinar de Investigação Médica (LIIM), Faculdade de Medicina, Universidade Federal de Minas Gerais, Avenida Prof. Alfredo Balena 190, Sala 281, 30130-100 Belo Horizonte, MG, Brazil; ^2^Laboratório de Neurofarmacologia, Instituto de Ciências Biológicas, Universidade Federal de Minas Gerais, Avenida Presidente Antônio Carlos 6627, 31270-901 Belo Horizonte, MG, Brazil; ^3^Laboratório de Neurobiologia, Instituto de Ciências Biológicas, Universidade Federal de Minas Gerais, Avenida Presidente Antônio Carlos 6627, 31270-901 Belo Horizonte, MG, Brazil; ^4^Departamento de Neurologia e Neurocirurgia, Santa Casa de Belo Horizonte Hospital, Avenida Francisco Sales 1111, 30150-221 Belo Horizonte, MG, Brazil

## Abstract

Cognitive impairment and depressive symptoms are of great interest in Parkinson's disease (PD), since they are very common and lead to increased disability with poor quality of life. Inflammatory mechanisms have been implicated in PD and its nonmotor symptoms. In the current pilot study, we aimed to evaluate plasma levels of chemokines in PD patients and to analyze the putative association of chemokines with depressive symptoms and cognitive performance. We hypothesized that higher chemokines levels are associated with worse cognitive performance and increased depressive symptoms in PD. For this purpose, 40 PD patients and 25 age- and gender-matched controls were subjected to a clinical evaluation including cognitive and mood tests. Peripheral blood was drawn and plasma levels of CCL2/MCP-1, CCL11/eotaxin, CCL24/eotaxin-2, and CXCL10/IP-10 were measured by enzyme-linked immunosorbent assay. PD patients and control individuals presented comparable plasma concentrations of all the evaluated chemokines. In PD patients, CXCL10/IP-10 plasma levels correlated positively with Hoehn and Yahr staging scale. In addition, the higher CXCL10/IP-10 levels, the worse performance on cognitive tests. Although there was no significant difference between PD patients and control individuals regarding chemokines levels, our preliminary results showed that CXCL10/IP-10 may be associated with cognitive status in PD.

## 1. Introduction

Parkinson's disease (PD) is the second most common neurodegenerative disorder worldwide. PD is characterized by the progressive loss of dopaminergic neurons of the* substantia nigra pars compacta* (SNpc) and the presence of alpha-synuclein intraneuronal inclusions called* Lewy* bodies in the remaining neurons [1]. It is well known that genetic mutations can cause familial parkinsonism, but only 10% of PD cases have a clear genetic origin. The etiopathogenesis of PD remains inconclusive in the great majority of cases [2]. Among several proposed causes of neuronal death in PD, mitochondrial dysfunction, oxidative stress, environmental toxins, and immune/inflammatory responses may be relevant candidates. The contribution of neuroinflammation (i.e., microglia activation, leukocytes infiltration, and increased levels of inflammatory mediators) to the pathophysiology of PD was first described in postmortem studies [3, 4]. Epidemiological, genetic, and immunological studies in humans and animal models have shown that not only neuroinflammation but also peripheral inflammatory changes may contribute to PD onset and development [5]. Lately, it has been shown that peripheral inflammatory and immune changes described in PD may be associated with some of the clinical signs, especially nonmotor symptoms, experienced by PD patients [6–9].

Chemokines are interesting molecular candidates to be studied in PD. Chemokines are chemotactic cytokines; that is, they attract and activate immune and nonimmune cells. For instance, they act as immune mediators, regulating leukocyte infiltration into the brain during inflammatory or infectious diseases [10]. A range of studies has also suggested that besides the well-established role in the immune system, chemokines and their receptors may also play an important role in the central nervous system (CNS). Chemokines and their receptors may modulate neurotransmitter release, regulating synaptic transmission, and cell survival. Guyon and colleagues demonstrated that the injection of CCL2/MCP-1 (monocyte chemotactic protein 1) onto dopaminergic neurons in the SNpc of rats increased cell excitability, dopamine release, and related locomotor activity [11]. Therefore, chemokines may represent a new class of neuromodulators, especially in dopaminergic neurons, potentially representing new targets for the treatment of PD. In addition, one post mortem study found that, despite the loss of dopaminergic neurons, the SNpc of PD patients exhibited increased levels of CXCR4 and its ligand CXCL12/SDF-1 (stromal cell-derived factor 1) in comparison with controls. Experiments in 1-methyl-4-phenyl-1,2,3,6-tetrahydropyridine- (MPTP-) induced PD mice confirmed these results, showing that MPTP produced a time-dependent upregulation of CXCR4 that preceded the loss of dopaminergic neurons [12]. Genetic studies also suggested the involvement of chemokines in PD. For instance, a single nucleotide polymorphism of the CXCL8/interleukin- (IL-) 8 A-251T gene was associated with PD in the Irish population [13]. Changes in the peripheral levels of chemokines such as CCL5/RANTES (acronym for regulated on activation, normal T cell expressed and secreted), CCL2/MCP-1, CCL3/MIP-1*α* (macrophage inflammatory protein-1 *α*), and CXCL8/IL-8 have been demonstrated in PD in comparison with controls [14, 15]. The dysregulation of chemokines is described in several neuropsychiatric disorders such as major depression [16], bipolar disorder [17], schizophrenia [18], obsessive-compulsive disorder [19], and Alzheimer's disease (AD) [20].

Although PD is largely characterized by specific motor signs, nonmotor symptoms are also frequent and disabling. Among these, cognitive impairment and depressive states are of great interest since they are common, progress as the disease advances, and lead to increased disability with poor quality of life [21–23]. Chemokines may influence functions such as mood, behavior, and cognition, possibly related to their neuromodulatory and/or direct neurotransmitter-like effects, regulation of neuroendocrine axes, control of the blood-brain barrier (BBB) permeability, and regulation of neurogenesis (for a review, see [24]). In the current study, we aimed to evaluate the plasma levels of chemokines in PD patients and to investigate the putative association of their levels with depressive symptoms and performance in cognitive tests. We hypothesized that higher chemokines levels are associated with worse cognitive performance and more depressive symptoms in PD.

## 2. Material and Methods

### 2.1. Subjects

This study included 40 PD patients diagnosed according to the United Kingdom PD Brain Bank criteria [25] and a group of 25 healthy controls matched by age, gender, body mass index (BMI), and educational level. Patients were recruited from the Outpatient Movement Disorders Clinic of the “Santa Casa de Belo Horizonte” Hospital, Belo Horizonte, Brazil. Controls were recruited from the local community. Participants were excluded if they had undergone previous neurosurgery or if they had any other neurological disorder and/or cognitive decline (i.e., delirium or dementia), significant sensory impairment, and infectious or autoimmune diseases in activity in the previous four weeks. In addition, individuals who had used corticosteroids, anti-inflammatories, or antibiotics in the four weeks prior to the study were excluded. All subjects provided written informed consent before admission to the study. The Research Ethics Committee of the Universidade Federal de Minas Gerais, Brazil, approved this study.

### 2.2. Clinical Evaluation

All patients were evaluated with the Unified Parkinson's Disease Rating Scale (UPDRS) [26] which assesses different signs and symptoms of PD. The UPDRS scores were obtained in the “*on*” state of the disease. The modified Hoehn and Yahr staging scale (HY) was used to establish the stage of PD [27]. The modified Schwab and England activities of daily living (ADL) scale (S&E) were used to assess daily routine of PD patients [26].

All individuals were subjected to cognitive examination which included the Mini-Mental State Examination (MMSE) [28] adapted for the Brazilian elderly population. MMSE is a brief test for cognitive screening, comprising items from different domains such as orientation, attention, memory, and language. Since impairment in executive functioning is the most common cognitive deficit in PD patients, the Frontal Assessment Battery (FAB) was also used [29]. FAB is a brief assessment tool that evaluates executive functioning and consists of six subtests exploring cognitive processes related to the frontal lobes: conceptualization mental flexibility, motor programming, sensitivity to interference, inhibitory control, and environmental autonomy. In each subtest, scores range from 0 (worst) to 3 (best). The total FAB score is calculated by the sum of the scores of each of the six subtests. In addition, all participants were evaluated using Beck's Depression Inventory (BDI), a self-rating instrument for depressive symptoms comprising 21 items, each one ranging from 0 to 3, according to the severity of symptoms [30]. BDI has been validated as a tool for depression screening and diagnosis in PD.

### 2.3. Chemokines Assessment

Ten milliliters of blood were drawn by venipuncture in vacuum tubes containing heparin (Vacuplast, Huangyn, China) on the same day of the clinical assessment. In order to rule out any confounding factors caused by circadian rhythm, all samples were collected at the same time of the day, between 14 and 16 h. The whole blood samples were kept at room temperature and used within 2 h after having been drawn. These samples were then centrifuged at 3,000 g for 10 min, 4°C, twice. The plasma was collected and stored at −70°C until assayed. The samples were thawed and excess of proteins was removed by acid/salt precipitation as routinely performed in our laboratory [17, 31]. Briefly, we mixed equal volume of plasma and 1.2% trifluoroacetic acid/1.35 mol/L NaCl. The plasma/acid trifluoroacetic mixture was left at room temperature for 10 minutes. Afterward, the samples were centrifuged for 5 minutes at 10,000 rpm. We then adjusted the supernatants for salt content (0.14 mol/L sodium chloride and 0.01 mol/L sodium phosphate) and pH (7.4), for the determination of chemokines concentration.

Plasma levels of CCL2/MCP-1, CCL11/eotaxin, CCL24/eotaxin-2, and CXCL10/IP-10 (interferon gamma-induced protein 10) were measured by Enzyme-Linked Immunosorbent Assay (ELISA) according to the procedures supplied by the manufacturer (DuoSet, R&D Systems, Minneapolis, MN, USA). Concentrations are expressed as pg/mL. Lower detection limits for all analyzed chemokines were 10 pg/mL.

### 2.4. Statistical Analysis

Association between dichotomous variables was assessed with Fisher's exact test. All variables were tested for Gaussian distribution by the Shapiro-Wilk normality test. Two groups (patients versus controls) were compared by Mann-Whitney or Student's *t* tests when nonnormally or normally distributed, respectively. Spearman's correlation analyses were performed to examine the relationship between clinical variables and plasma levels of chemokines. All statistical tests were two-tailed and were performed using a significance level of *α* = 0.05. Statistical analyses were performed using SPSS software version 16.0 (SPSS Inc., Chicago, IL, USA).

## 3. Results

### 3.1. Sociodemographic and Clinical Results

Demographic and nonmotor features of both groups are shown in Table 1. PD patients' clinical data are given in Table 2. PD patients presented a worse performance in the MMSE in comparison with controls (*Z* = −3,325; *P* = 0.001). There was no difference between PD and control individuals regarding total FAB performance. Nonetheless, the analysis of the subtests demonstrated that PD patients presented a lower score in programming (*Z* = −2,107; *P* = 0.04). In addition, BDI score was higher in PD patients compared to controls (*Z* = −3,528; *P* < 0.001).

Regarding PD patients, there was a negative correlation between total FAB score and age (*ρ* = −0.445, *P* = 0.005). As expected, higher educational level was associated with better performance in both cognitive tests, MMSE and FAB (*ρ* = 0.429, *P* = 0.006, and *ρ* = 0.463, *P* = 0.003, resp.). BDI score correlated positively with UPDRS total score and HY stage (*ρ* = 0.421, *P* = 0.010 and *ρ* = 0.440, *P* = 0.006, resp.) and negatively with S&E scale (*ρ* = −0.457, *P* = 0.004). Considering control subjects, total FAB performance correlated negatively with age and BDI score (*ρ* = −0.677, *P* < 0.001 and *ρ* = −0.534, *P* = 0.006, resp.). Here again, educational level correlated positively with MMSE and FAB scores (*ρ* = 0.790, *P* < 0.001 and *ρ* = 0.771, *P* = 0.001, resp.).

### 3.2. Plasma Levels of Chemokines

**Figure 1:**
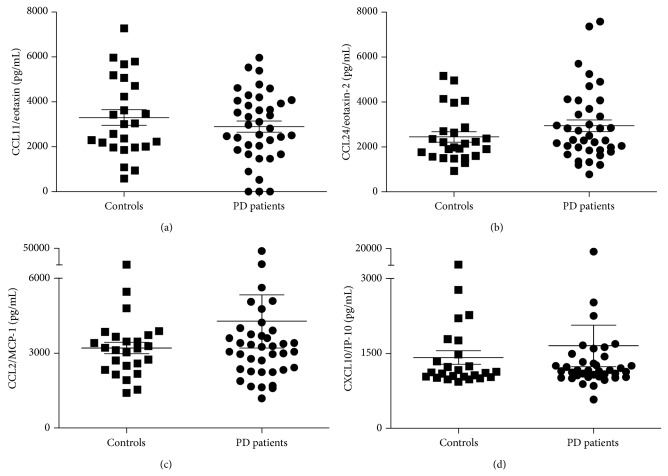
Control individuals and Parkinson's disease (PD) patients did not differ regarding plasma levels of the evaluated chemokines CCL11/eotaxin (a), CCL24/eotaxin-2 (b), CCL2/MCP-1 (c), and CXCL10/IP-10 (d). The figure shows mean and standard deviation of the mean (SEM). IP-10: interferon gamma-induced protein-10; MCP-1: monocyte chemotactic protein 1; PD: Parkinson's disease.

As shown in Figure 1, there was no significant difference between PD patients and controls regarding plasma levels of the evaluated chemokines CCL11/eotaxin (*t* = −0.967; *P* = 0.34, Student's *t* test), CCL24/eotaxin-2 (*Z* = −1,300; *P* = 0.19, Mann-Whitney test), CXCL10/IP-10 (*Z* = −0.035; *P* = 0.97, Mann-Whitney test), and CCL2/MCP-1 (*Z* = −0.148; *P* = 0.88, Mann-Whitney test).

**Figure 2:**
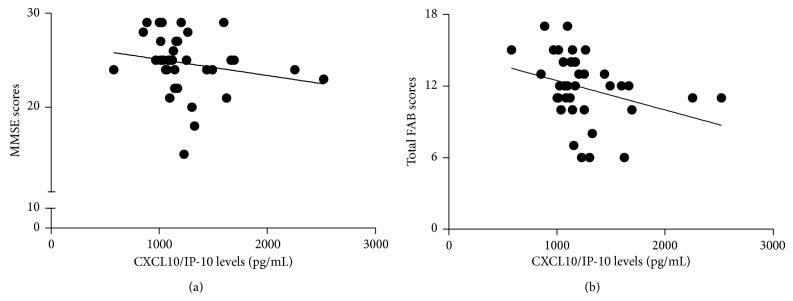
Among Parkinson's disease (PD) patients, plasma levels of CXCL10/IP-10 were inversely associated with MMSE (a; *ρ* = −0.395, *P* = 0.016) and total FAB scores (b; *ρ* = −0.458, *P* = 0.004). IP-10: interferon gamma-induced protein-10; MMSE: Mini-Mental State Examination; FAB: Frontal Assessment Battery.

Circulating CXCL10/IP-10 levels were associated with PD progression since CXCL10/IP-10 plasma concentration correlated positively with HY staging scale (*ρ* = 0.366, *P* = 0.026). Moreover, among PD patients increased CXCL10/IP-10 levels were associated with worse performance in cognitive tests; that is, CXCL10/IP-10 levels correlated negatively with MMSE (*ρ* = −0.395, *P* = 0.016) and FAB (*ρ* = −0.458, *P* = 0.004) scores (Figure 2). More specifically, increased CXCL10/IP-10 levels were associated with decreased mental flexibility (*ρ* = −0.439, *P* = 0.007) and inhibitory control (*ρ* = −0.365, *P* = 0.026). It is worth mentioning that the correlation between CXCL10/IP-10 levels and total FAB score remained significant even after Bonferroni's correction. We found no correlation between plasma levels of chemokines and BDI or S&E scores. In addition, we found no association between chemokines levels and clinical or demographic data in control subjects.

## 4. Discussion

This report demonstrated that PD patients and control individuals present comparable plasma concentrations of the chemokines CCL2/MCP-1, CCL11/eotaxin, CCL24/eotaxin-2, and CXCL10/IP-10. Interestingly, increased CXCL10/IP-10 levels were associated with PD progression and worse performance in cognitive tests.

Only few studies have evaluated circulating levels of chemokines in PD and the results are inconclusive. Our work group has already measured serum levels of chemokines in an independent cohort of PD patients, finding no significant difference between PD patients and age- and gender-matched controls [31], corroborating the current data. Rentzos and colleagues [14] evaluated serum levels of CCL5/RANTES in 41 PD patients and 19 controls. PD patients presented higher levels of CCL5/RANTES in comparison with controls, and circulating levels of CCL5/RANTES positively correlated with UPDRS III scores. Remarkably, untreated patients (*n* = 20) showed higher levels of CCL5/RANTES than control and treated PD groups [14], indicating that PD-related drugs may interfere with chemokines levels.

Another study has demonstrated that basal and lipopolysaccharide- (LPS-) induced levels of the chemokines CCL2/MCP-1, CCL3/MIP-1*α*, and CXCL8/IL-8 and basal levels of CCL5/RANTES were significantly higher in PD patients than in controls. In addition, chemokines levels were associated with PD severity, since they correlated positively with UPDRS III scores and HY stages [15]. It is worth mentioning that, although all patients were taking anti-parkinsonian drugs, as well as in our study, they used a different strategy to assess peripheral levels of chemokines; that is, they measured the levels of chemokines produced by peripheral blood mononuclear cells instead of serum/plasma quantifications [15].

To the best of our knowledge, the current study is the first to describe the association between peripheral chemokines levels and cognitive performance in PD. A few studies have assessed the association between chemokines and nonmotor symptoms in PD. One recent study evaluated the association between nonmotor symptoms and chemokines assessed in the cerebrospinal fluid (CSF) of PD patients [32]. With this purpose, CCL11/eotaxin, CXCL-10/IP-10, CCL2/MCP-1, and CCL4/MIP-1*β* levels were measured in the CSF of 87 PD patients (16 with dementia diagnosis) and 33 controls. Corroborating our results, there was no difference between groups for any of the measured CSF chemokines. CCL2/MCP-1 levels correlated with symptoms of depression, as assessed by the Hospital Anxiety and Depression Scale, and UPDRS motor score. FACIT-fatigue score correlated negatively with CXCL10/IP-10 and CCL2/MCP-1 levels; that is, the higher the levels of chemokines the more severe the fatigue symptoms. They did not find any significant correlation between cognitive performance as assessed by the MMSE and the evaluated chemokines [32]. Another study measured CX3CL1/fractalkine levels in the CSF of 126 PD patients in comparison with multiple systems atrophy patients (*n* = 32), AD patients (*n* = 50), and age-matched controls (*n* = 137) [33]. Again, there was no significant difference between groups regarding chemokine CSF levels. CSF fractalkine/amyloid-*β* ratio increased significantly with increasing UPDRS scores, HY stage, and being a putative biomarker for PD severity. In an independent cohort comprising 39 PD patients, CX3CL1/fractalkine alone correlated with the clinical progression of PD [33]. Interestingly, an experimental study showed that this chemokine is neuroprotective in MPTP-induced PD mice [34].

We found that CXCL10/IP-10 levels were associated with PD progression. The chemokine CXCL10/IP-10 has already been associated with PD pathophysiology, since IL-1*β*-induced CXCL10/IP-10 protein expression was potentiated by coexposure to *α*-synuclein in human A172 astroglial cells [35]. The chemokine CXCL10/IP-10 has also been associated with cognitive status in AD, the prototype cognitive disorder. CSF CXCL10/IP-10 concentration was significantly increased in patients with mild cognitive impairment and mild AD in comparison with controls, but not in patients with severe AD. There was a significant positive correlation between MMSE score and CSF CXCL10/IP-10 levels [36]. Nonetheless, the same authors showed that serum levels of CXCL10/IP-10 were not increased in mild cognitive impairment and AD regardless of the stage of the disease. In addition, no correlation between serum CXCL10/IP-10 levels and MMSE scores or the duration of the disease was found [37]. These observations suggest that CSF CXCL10/IP-10 levels increase may be restricted to an early stage of the disease, when proinflammatory events are thought to play a more relevant role in its pathogenesis [37]. Since our sample is composed by patients with moderate to advanced PD (mean age = 68 years; mean disease duration = 5.5 years; mean total UPDRS score = 51.8; mean motor UPDRS score = 34.6), it is not possible to test this hypothesis, which deserves further investigation.

Limitations of our study include sample size and the fact that all patients were under anti-parkinsonian treatment that may influence the results. However, statistical analysis showed that the use of antiparkinsonian or other drugs did not influence chemokines levels in the present study. We also have to take into account that only peripheral blood levels of chemokines were evaluated, and they did not necessarily reflect what it is going on in the CNS. The parallel assessment of chemokines in the CSF would be interesting. By contrast, the strict exclusion criteria, the selection of controls with comparable age, gender, and BMI, and the analysis of clinical and inflammatory parameters together can be regarded as strengths of the study.

Although there was no significant difference between PD patients and control individuals regarding plasma levels of chemokines, our results showed that CXCL10/IP-10 levels may be associated with cognitive status in PD. Additional studies are needed in order to elucidate chemokines role in PD, mainly regarding cognitive impairment.

## Figures and Tables

**Table 1:** Demographic and nonmotor features of Parkinson's disease (PD) patients and control subjects.

	PD patients (*n* = 40)	Control subjects (*n* = 25)	*P* value
Gender (female/male)	13/27	6/19	0.58^a^
Age in years (mean ± SD)	68.71 ± 10.07	65.23 ± 8.75	0.20^b^
Body mass index in Kg/m^2^ (mean ± SD)	26.02 ± 3.73	27.64 ± 3.71	0.09^c^
Educational level in years (mean ± SD)	4.72 ± 2.87	6.72 ± 5.37	0.16^b^
MMSE [mean ± SD (median)]	**24.00 ± 3.99 (25)**	**27.00 ± 3.57 (29)**	**0.001** ^ b^
FAB [mean ± SD (median)]	11.49 ± 2.99 (12)	12.32 ± 3.67 (13)	0.32^c^
Conceptualization	1.23 ± 1.01 (1)	1.64 ± 1.11 (2)	0.12^b^
Mental flexibility	1.82 ± 1.10 (2)	2.08 ± 1.04 (2)	0.34^b^
Programming	**1.74 ± 0.91 (2)**	**2.24 ± 0.83 (2)**	**0.04** ^ b^
Sensitivity to interference	2.26 ± 0.94 (3)	1.84 ± 1.25 (2)	0.21^b^
Inhibitory control	1.41 ± 0.88 (1)	1.52 ± 1.09 (1)	0.73^b^
Environmental autonomy	3.00 ± 0.00 (3)	3.00 ± 0.00 (3)	1.00^b^
BDI [mean ± SD (median)]	**8.64 ± 7.58 (6)**	**2.76 ± 3.35 (1)**	**<0.001** ^ b^
Medication in use (frequency in %)			
Antihypertensive (%)	55.00	48.00	0.62^a^
Antidiabetic (%)	10.00	20.00	0.29^a^
Hypolipidemic (%)	10.00	24.00	0.17^a^
Levothyroxine (%)	10.00	4.00	0.64^a^
Antidepressants	20.00	12.00	0.51^a^

BDI: Beck Depression Inventory; FAB: Frontal Assessment Battery; MMSE: Mini-Mental State Examination; PD: Parkinson's disease; SD: standard deviation.

^a^Fisher's exact test; ^b^Mann-Whitney test; ^c^Student's *t* test.

**Table 2:** Clinical features of Parkinson's disease (PD) patients.

	PD patients (*n* = 40)
Length of illness in years [mean ± SD (range)]	5.45 ± 4.13 (0.4–18)
UPDRS [mean ± SD (range)]	51.82 ± 25.27 (11–105)
UPDRS I [mean ± SD (range)]	3.36 ± 2.96 (0–11)
UPDRS II [mean ± SD (range)]	14.08 ± 7.14 (2–31)
UPDRS III [mean ± SD (range)]	34.56 ± 18.43 (8–69)
HY [mean ± SD (range)]	2.44 ± 0.69 (1–4)
S&E in % [mean ± SD (range)]	77.95 ± 11.96 (50–100)
Medications in use [frequency (%)]	
Levodopa	37 (92.50)
Pramipexole	20 (50.00)
Entacapone	7 (17.50)
Amantadine	11 (27.50)

HY: Hoehn and Yahr staging scale; PD: Parkinson's disease; SD: standard deviation; S & E: Schwab and England activities of daily living scale; UPDRS: Unified Parkinson's Disease Rating Scale.
